# Supplementary material for: Is the Contralateral Delay Activity (CDA) a robust neural correlate for Visual Working Memory (VWM) tasks? A reproducibility study
Source: Psychophysiology. 2022 Sep 19;60(2):e14180. doi: 10.1111/psyp.14180 (PMC10078237; doi:10.1111/psyp.14180)
Supplement: Supplementary file 1 — Appendix S1 Supporting Information [file PSYP-60-0-s001.zip › PSYP_14180_CDA_Reproducibility_Roy2022_SUPPMAT_FINAL.docx]

# 8 Supplementary Material

**[Figure 18]**

**[Figure 19]**

**[Figure 20]**

**[Figure 21]**

**[Figure 22]**

**[Figure 23]**

**[Figure 24]**

**[Figure 25]**

# Figure Captions – Supplementary Material

Figure 18: Top 5 and Bottom 5 from Villena-Gonzalez, 2019. The graphs show the CDA averaged across trials for 3 different conditions for a total of 10 participants. The *Top 5*, shown in blue, represents the 5 participants with the highest performance while the *Bottom 5*, in orange, represents the 5 participants with the lowest performance.

Figure 19: Top 5 and Bottom 5 from Adam, 2018. The graphs show the CDA averaged across trials for 3 different conditions for a total of 10 participants. The *Top 5*, shown in blue, represents the 5 participants with the highest performance while the *Bottom 5*, in orange, represents the 5 participants with the lowest performance.

Figure 20: Top 5 and Bottom 5 from Feldmann-Wusterfel 2020. The graphs show the CDA averaged across trials for 3 different conditions for a total of 10 participants. The *Top 5*, shown in blue, represents the 5 participants with the highest performance while the *Bottom 5*, in orange, represents the 5 participants with the lowest performance.

Figure 21: Top 5 and Bottom 5 from Balaban 2019 Exp. 2. The graphs show the CDA averaged across trials for 3 different conditions for a total of 10 participants. The *Top 5*, shown in blue, represents the 5 participants with the highest performance while the *Bottom 5*, in orange, represents the 5 participants with the lowest performance.

Figure 22: CDA Amplitude vs Performance - Feldmann-Wüstefeld, 2020

Figure 23: CDA Amplitude vs Performance - Adam, 2018

Figure 24: Getting Started from Feldmann-Wüstefeld, 2020

Figure 25: README from Adam, 2018
