# Supplementary figures and images for: Is the Contralateral Delay Activity (CDA) a robust neural correlate for Visual Working Memory (VWM) tasks? A reproducibility study
Source: Psychophysiology. 2022 Sep 19;60(2):e14180. doi: 10.1111/psyp.14180 (PMC10078237; doi:10.1111/psyp.14180)

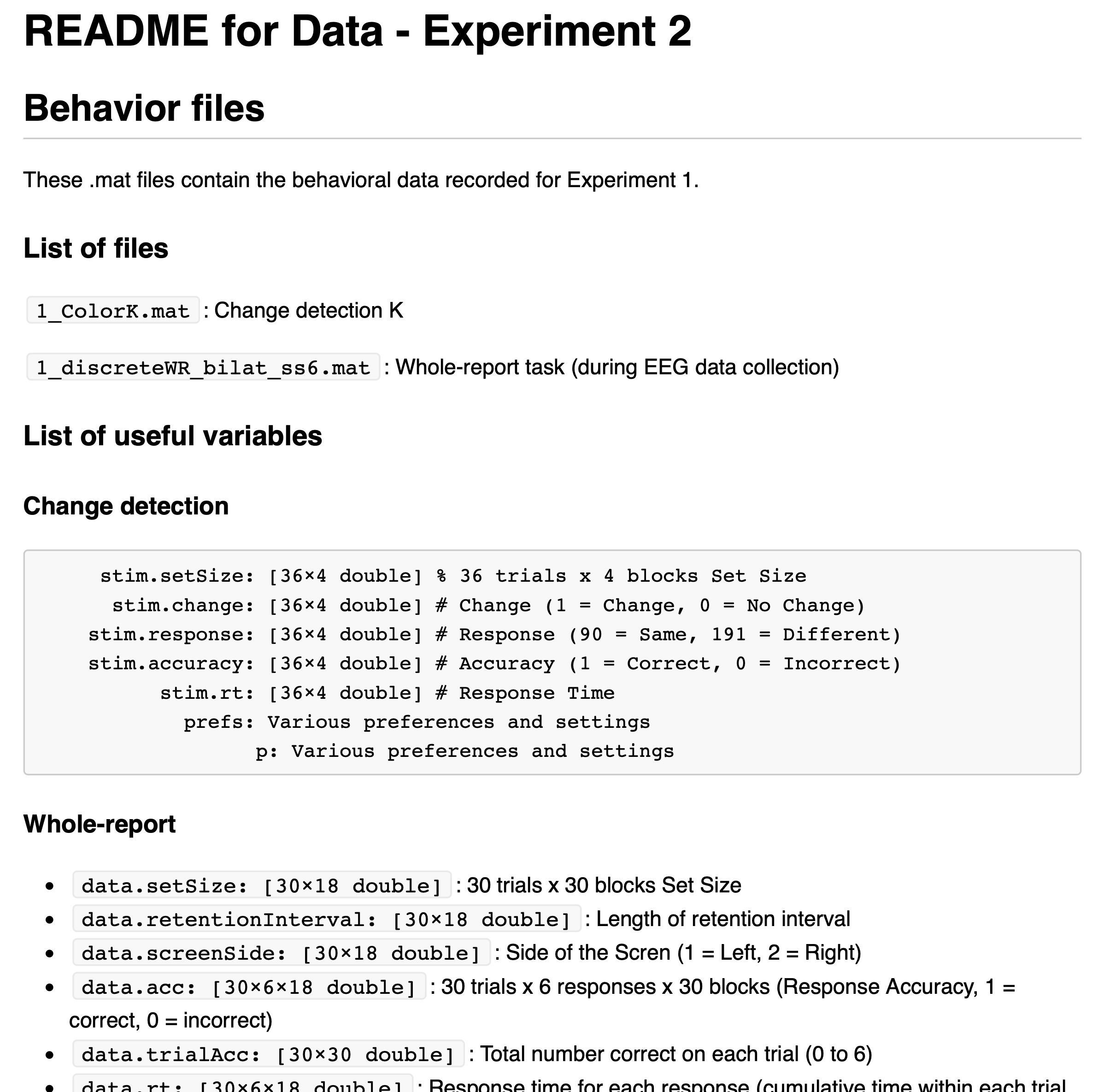

Supplement: Supplementary file 1 — Appendix S1 Supporting Information [file PSYP-60-0-s001.zip › PSYP_14180_A2018-ReadMe.png]

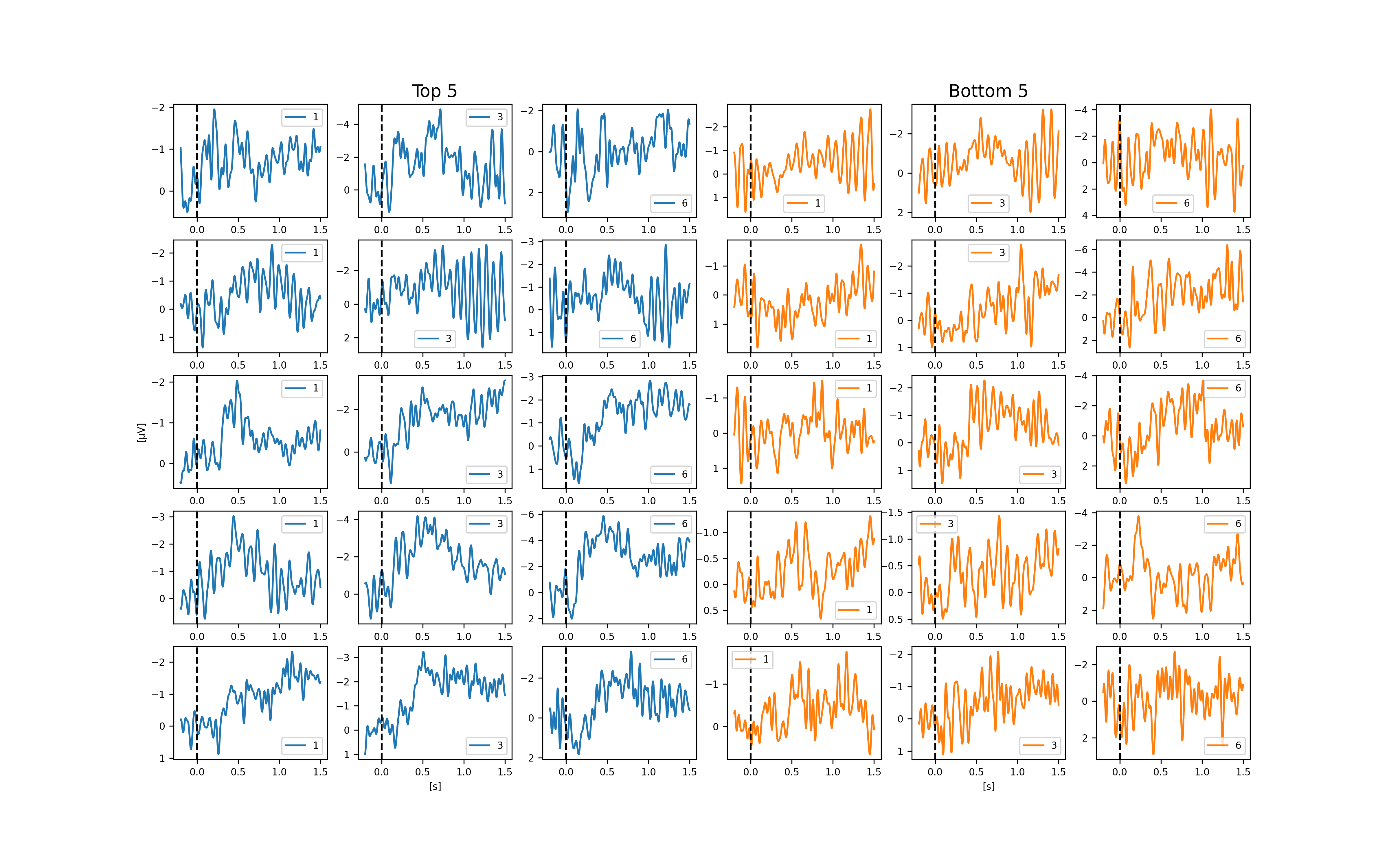

Supplement: Supplementary file 1 — Appendix S1 Supporting Information [file PSYP-60-0-s001.zip › PSYP_14180_Adam2018-Exp1_top5low5.png]

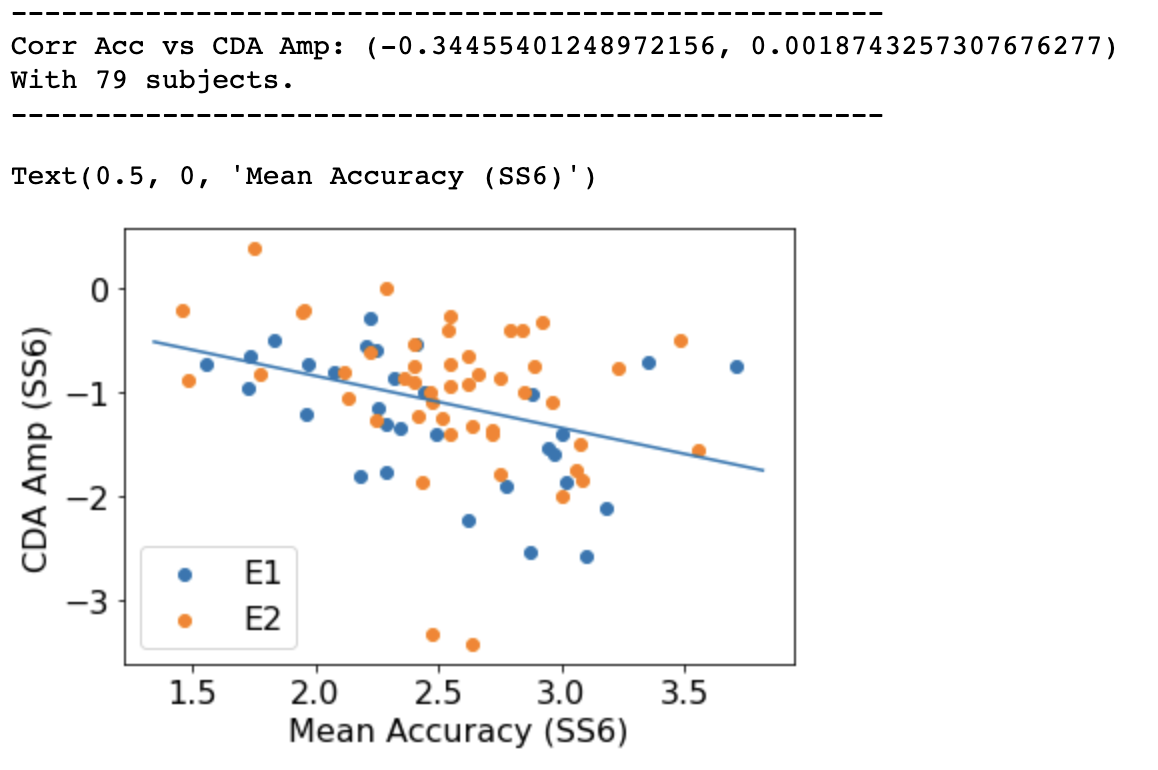

Supplement: Supplementary file 1 — Appendix S1 Supporting Information [file PSYP-60-0-s001.zip › PSYP_14180_Adam2018-KMeanCDAPerf.png]

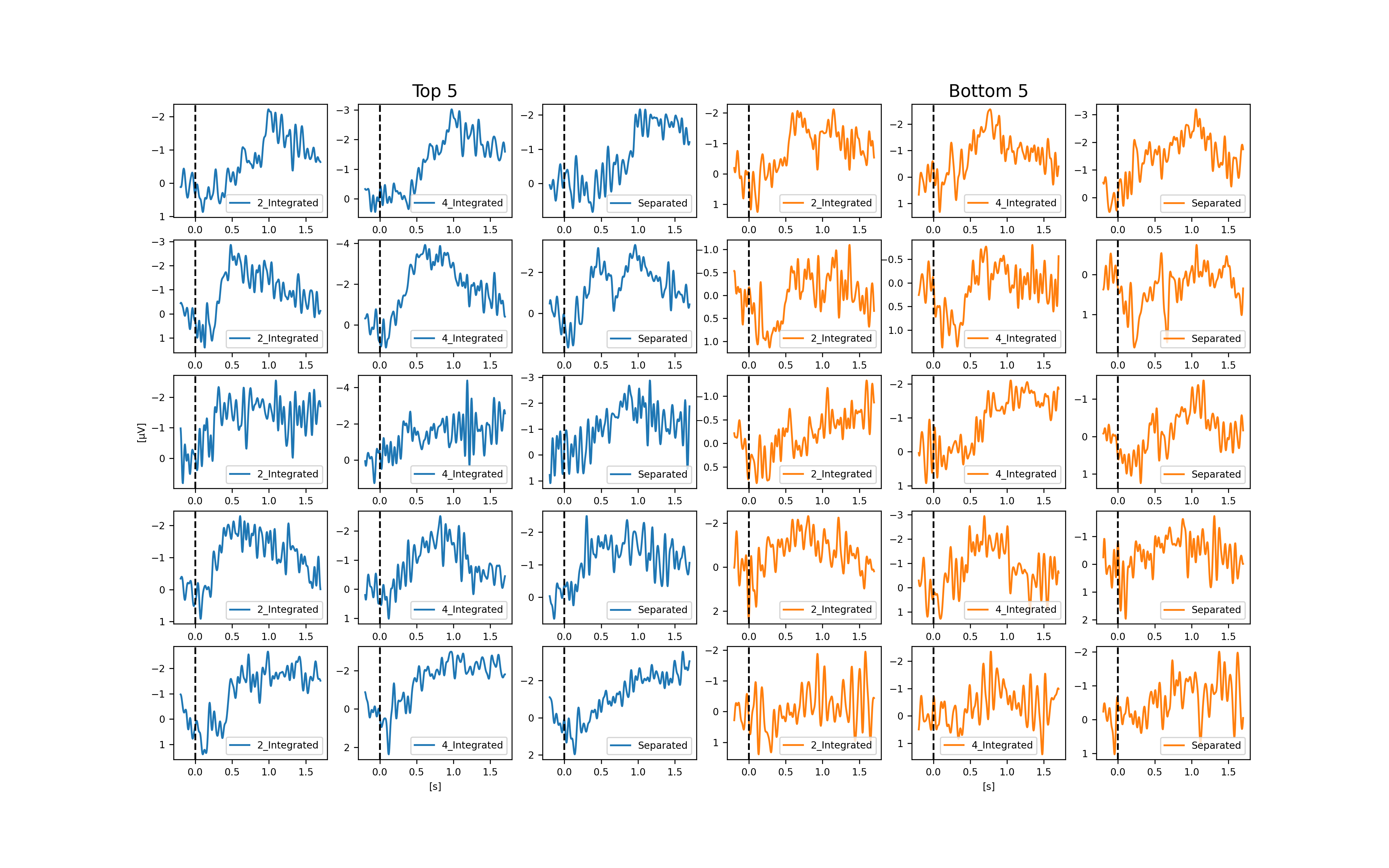

Supplement: Supplementary file 1 — Appendix S1 Supporting Information [file PSYP-60-0-s001.zip › PSYP_14180_Balaban2019-Exp2_top5low5.png]

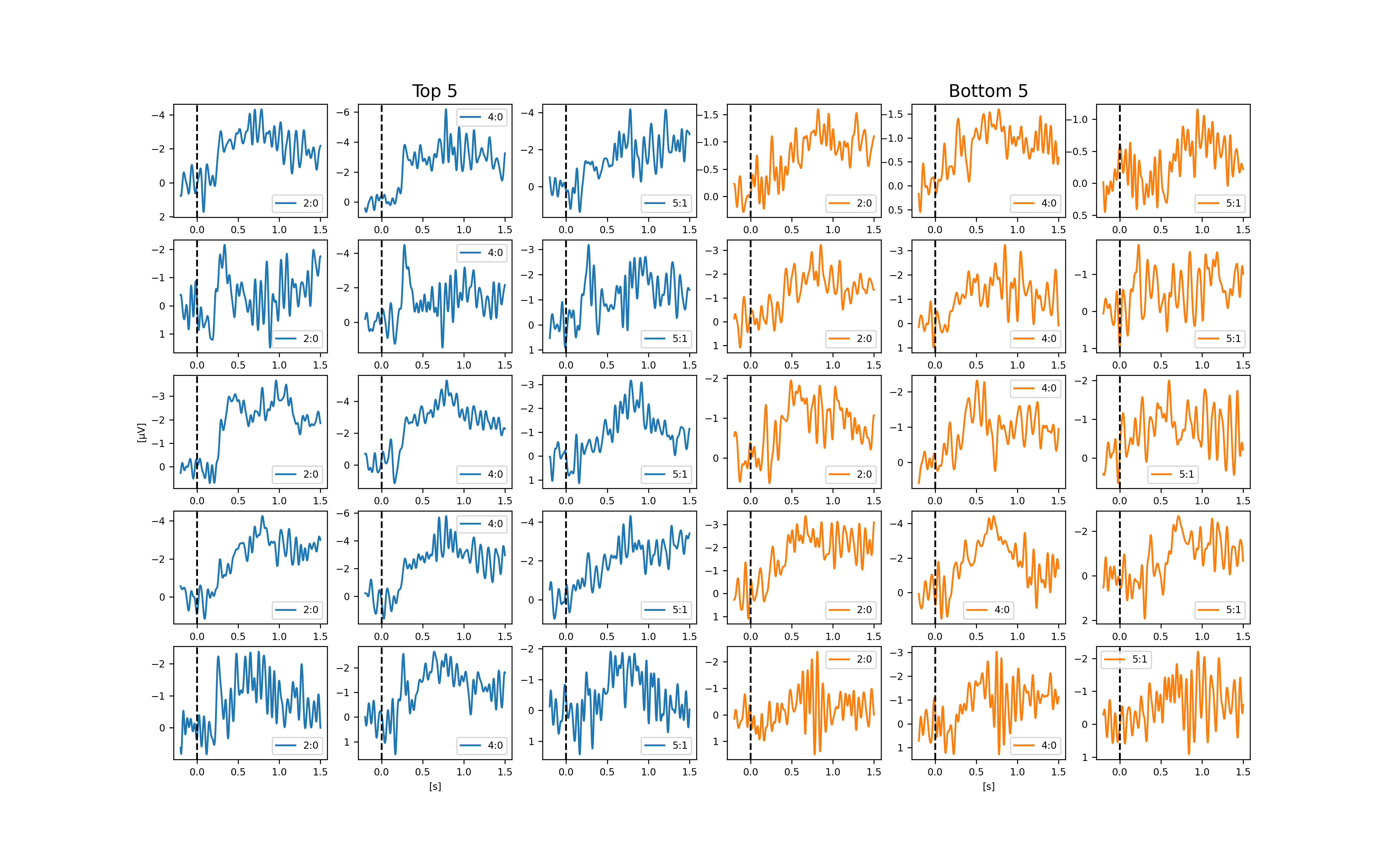

Supplement: Supplementary file 1 — Appendix S1 Supporting Information [file PSYP-60-0-s001.zip › PSYP_14180_Feldmann2020_top5low5.png]

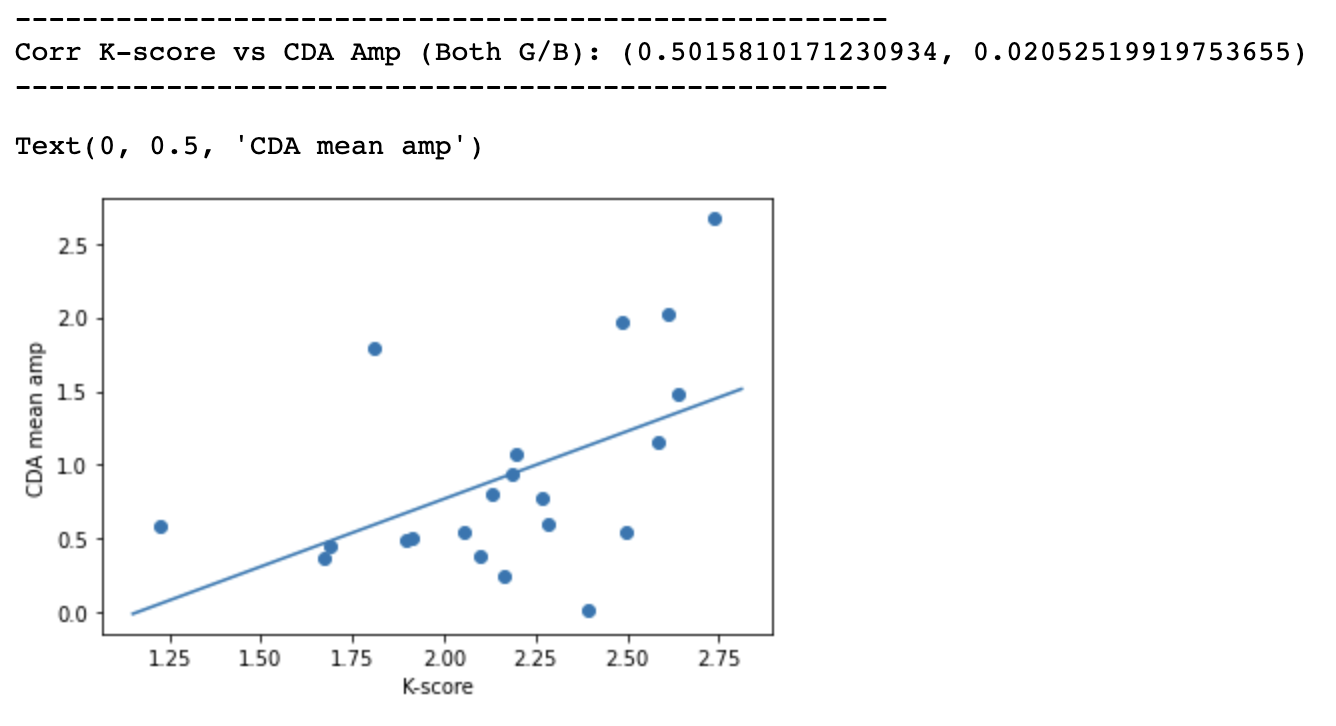

Supplement: Supplementary file 1 — Appendix S1 Supporting Information [file PSYP-60-0-s001.zip › PSYP_14180_Feldmann2020-KMeanCDAPerf.png]

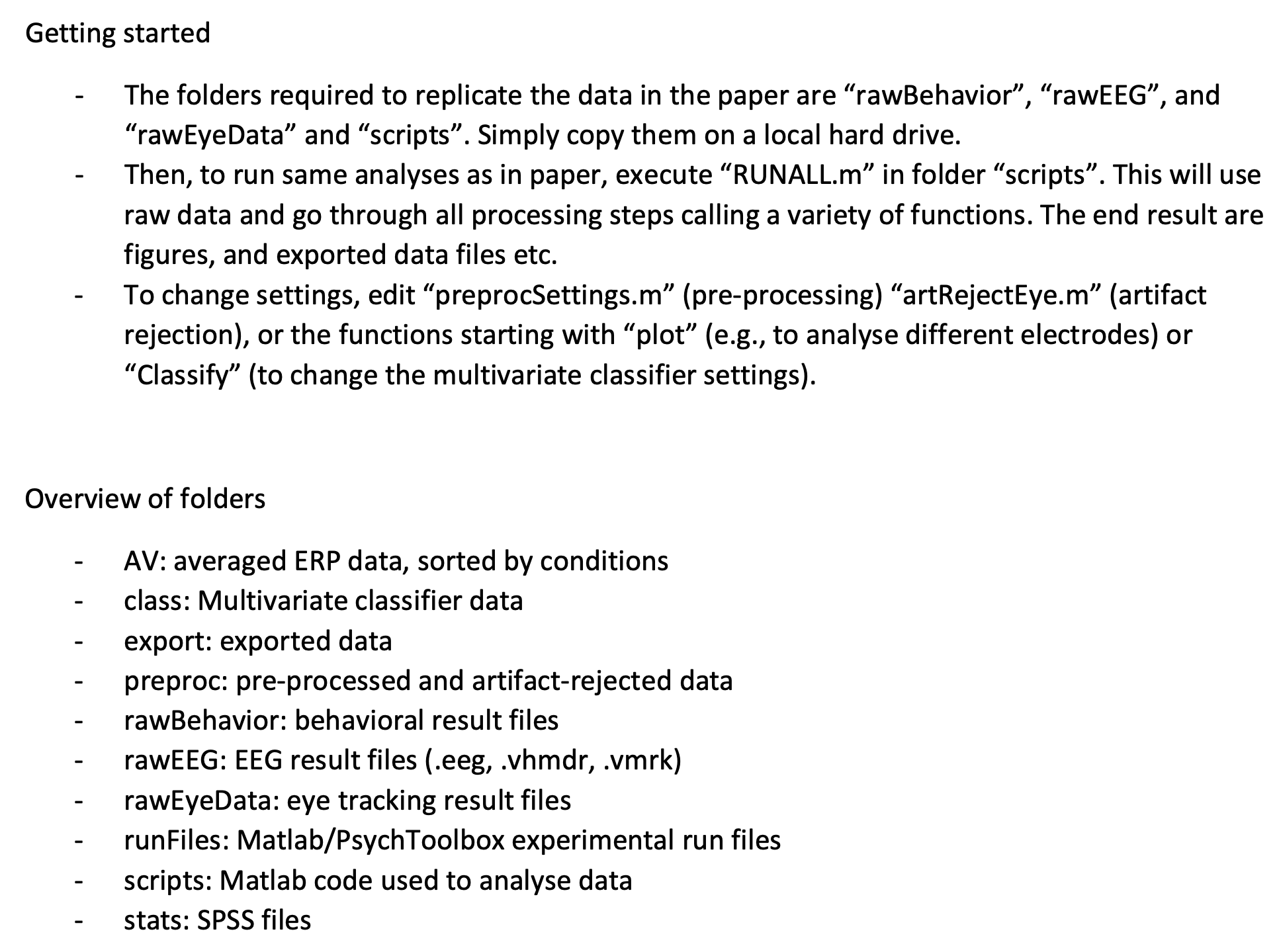

Supplement: Supplementary file 1 — Appendix S1 Supporting Information [file PSYP-60-0-s001.zip › PSYP_14180_FW2020-GettingStarted.png]

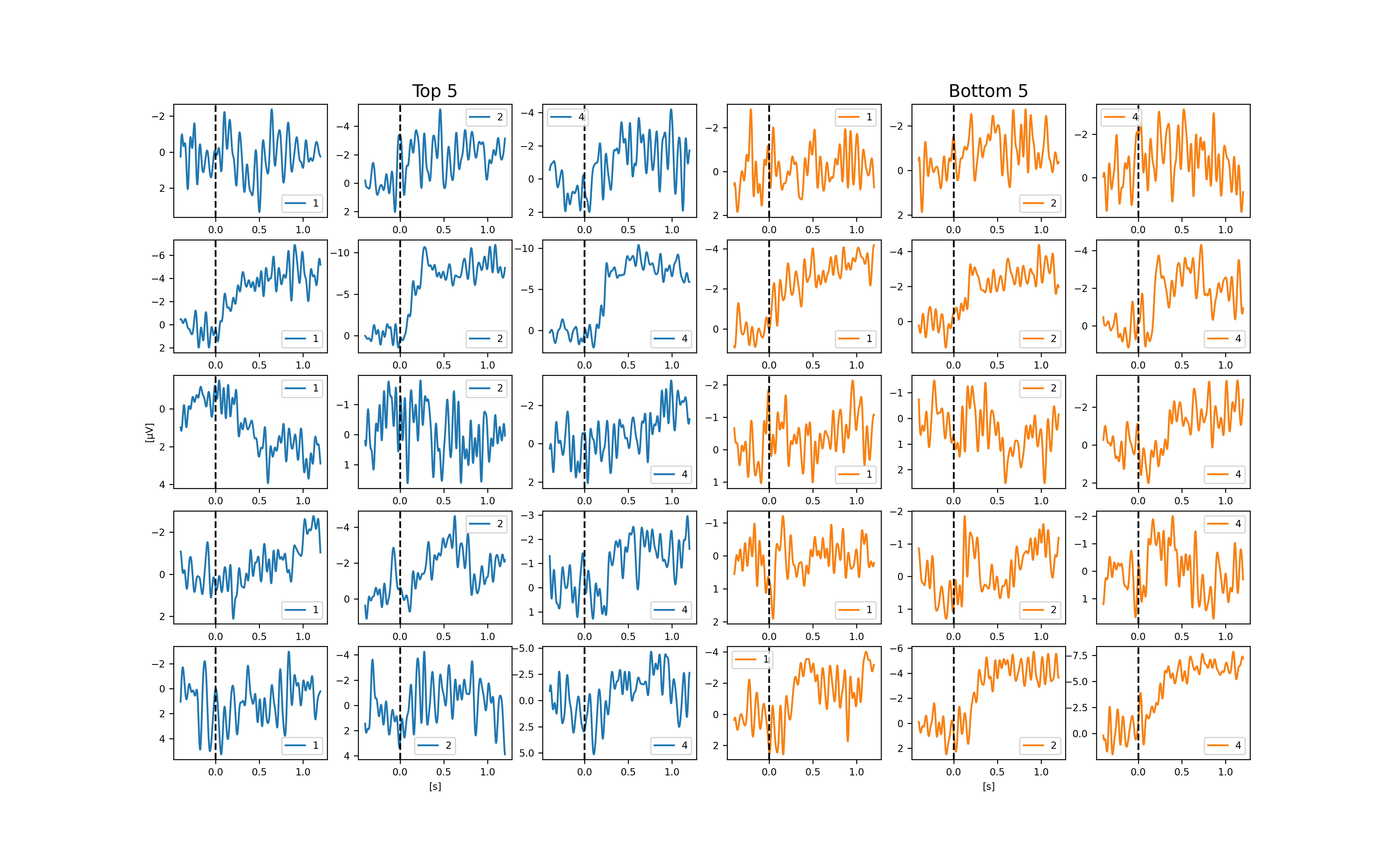

Supplement: Supplementary file 1 — Appendix S1 Supporting Information [file PSYP-60-0-s001.zip › PSYP_14180_villena2019_top5low5.png]
